# Ramadan Intermittent Fasting Is Associated with Changes in Circulating Proprotein Convertase Subtilisin/Kexin Type 9 (PCSK9) in Metabolically Healthy Obese Subjects

**DOI:** 10.3390/medicina58040503

**Published:** 2022-03-31

**Authors:** Hayder Hasan, Mohamed Madkour, Samir Awadallah, Mohamed Hassanein, Haitham Jahrami, MoezAlIslam Faris

**Affiliations:** 1 Department of Clinical Nutrition and Dietetics, College of Health Sciences/Research Institute for Medical and Health Sciences (RIMHS), University of Sharjah, Sharjah P.O. Box 27272, United Arab Emirates; haidarah@sharjah.ac.ae; 2 Department of Medical Laboratory Sciences, College of Health Sciences/Research Institute for Medical and Health Sciences (RIMHS), University of Sharjah, Sharjah P.O. Box 27272, United Arab Emirates; mmadkour@sharjah.ac.ae (M.M.); sawadallah@sharjah.ac.ae (S.A.); 3 Endocrine Department, Dubai Hospital, Dubai Health Authority, Dubai P.O. Box 7272, United Arab Emirates; mhassanein148@hotmail.com; 4 College of Medicine and Medical Sciences, Arabian Gulf University, Manama P.O. Box 26671, Bahrain; hjahrami@health.gov.bh; 5 Ministry of Health, Manama P.O. Box 12, Bahrain

**Keywords:** blood lipids, caloric restriction, cardiometabolic risk factors, intermittent fasting, lipid metabolism, obesity

## Abstract

*Background and Objectives:* Dietary modification is the principal approach to the management of hyperlipidemia in adults. Proprotein convertase subtilisin/kexin type 9 (PCSK9) is a key regulator of plasma cholesterol and a target for novel lipid-lowering pharmacotherapies. This study aimed to explore how circulating levels of PCSK9 changed during Ramadan intermittent fasting in metabolically healthy obese subjects. *Materials and Methods*: This cross-sectional study used convenience sampling to recruit 55 overweight and obese participants (22 females and 33 males) who observed Ramadan fasting. Body weight and composition, glucoregulatory factors, serum PCSK9 concentration, dietary intake, and physical activity were assessed 1 week before and at the end of Ramadan fasting. *Results:* The median (interquartile range) age was 35 (22) years, and body mass index was 30.2 (5.4). We found significant (*p* < 0.05) increases in serum levels of PCSK9, serum insulin, insulin resistance, and leptin at the end of Ramadan compared with pre-fasting levels. Significant (*p* < 0.05) reductions in body weight, waist circumference, systolic and diastolic blood pressure, total cholesterol, triglycerides, high-density lipoprotein cholesterol, and adiponectin were also observed at the end of Ramadan. *Conclusions:* Observing Ramadan fasting was associated with increased PCSK9 levels in metabolically healthy obese subjects. The complex relationships between PCSK9 and insulin resistance and dysregulation of adipokine secretion in relation to dietary and lifestyle modifications during Ramadan warrant further research.

## 1. Introduction

Intermittent fasting (IF) is receiving increased attention from scientific bodies [1]. A frequently investigated model of IF is time-restricted eating (TRE) or feeding, which includes Ramadan fasting [2]. Fasting during Ramadan (lunar month of 29–30 days) is mandatory for all healthy adult Muslims. Amid Ramadan, Muslims refrain from drinking and eating from dawn to sunset. For healthy individuals, fasting during Ramadan has beneficial health effects and can be a health-promoting practice [3]. However, fasting represents a significant deviation from usual daily life in terms of meal frequency, time, and composition [4], as well as sleeping times and patterns [5]. These changes ultimately affect appetite [6] and hormonal responses [7].

Various systematic reviews and meta-analyses, along with original research, have shown that Ramadan intermittent fasting (RIF) improves metabolic syndrome components, including triglycerides (TG), high-density lipoprotein cholesterol (HDL), fasting glucose (FG), waist circumference (WC), and blood pressure [8]; visceral adiposity [9]; inflammatory and oxidative stress markers [10,11]; body weight [12]; body fat [13]; glucometabolic [14] and cardiometabolic risk factors [15]; and liver function tests [16]. This fasting also impacts glucometabolic markers (e.g., adiponectin, leptin, insulin resistance, and serum insulin levels) [7,14]. Furthermore, a meta-analysis indicated that RIF may be associated with a moderate improvement in lipid components—especially HDL [8,15]. Other meta-analyses investigating the impact of TRF and intermittent caloric restriction (including RIF) indicated that hypocaloric and normocaloric IF may aid in improving the blood lipids in obese and dyslipidemic women and men by increasing HDL-C levels and reducing total cholesterol (TC), TG, and low-density lipoprotein cholesterol (LDL) [17]. However, another meta-analysis found that TRF did not have a significant effect on blood lipids in adults with excess weight [18]. These differing reports regarding the impact of IF—including TRF and RIF—may be ascribed to the variability in the food quality and quantity consumed, as well as changes in body weight at the end of Ramadan [19].

Proprotein convertase subtilisin/kexin type 9 (PCSK9)—a blood protein synthesized by the liver—works as an inhibitor of the pathway of the LDL-C receptor (LDLR) by targeting receptors in the lysosomal degradation pathway [20]. Levels of circulating PCSK9 are associated with LDL-C [21], as well as with insulin resistance and TG to a lesser extent [22]. Therefore, elevated levels of PCSK9 may result in escalated cardiovascular disease (CVD) risk [23], especially among type 2 diabetic patients [24]. Such findings have prompted research into possible ways to combat high levels of circulating PCSK9, including pharmacological and non-pharmacological approaches. The use of PCSK9 inhibitors is increasingly popular for dyslipidemic people and those at high risk of developing CVD who do not respond to statin treatment. Furthermore, recent studies have proposed a direct association between PCSK9, inflammation, and the potential inhibitory effects of anti-inflammatory nutritional factors against this enzyme [25].

As a non-pharmacological approach, dietary modification was found to have a marked effect on plasma PCSK9 levels [26]. In addition, RIF has been associated with significant changes in blood lipids [8,14], which may affect circulating levels of PCSK9 However, there is a paucity of studies focused on changes in serum PCSK9 concentrations following RIF. The increased attention directed to the impact of intermittent fasting [27]—including RIF—on cardiometabolic health justifies the exploration of changes in the circulating levels of PCSK9 among metabolically healthy obese people observing Ramadan. Based on the available literature concerning the positive impacts of RIF on body weight, body composition, and metabolic markers, we hypothesized that practicing RIF would decrease PCSK9 levels in metabolically healthy obese subjects.

## 2. Methods and Materials

### 2.1. Study Design

A prospective study design was followed during Ramadan (from June 2016 to July 2016, corresponding to the month of Ramadan in the lunar calendar), where the daily fasting period was about 15 h. Data collection started one week before (baseline or pre-fasting) Ramadan, and after completing 28–30 days (end of Ramadan) of Ramadan. During Ramadan, fasting Muslims abstain from foods and drinks (including water) and do not smoke from dawn to sunset. We compared the studied variables for each participant before and at the end of Ramadan, meaning that each participant served as their own control. Participants did not receive any nutritional recommendations or physical activity advice at any stage during this study. The Islamic laws of Ramadan excuse females from fasting during Ramadan during their menstrual period; therefore, the fasting days for female participants were about 23–25 days.

### 2.2. Subjects

This observational study included a convenience sample of adults from the United Arab Emirates (UAE), including expatriates from Jordan, Palestine, Syria, Egypt, and Sudan who were residing in Sharjah. Potential participants were invited to enroll in this study through social media, the hospital bulletin, and personal communications. Announcement of the inclusion criteria (healthy, male/female, without predetermined chronic disease or health problem, not enrolled in a weight-reducing regime, or having undergone bariatric surgery in the last 6–9 months) was disseminated via social media and personal contacts. Those who were interested in the study contacted us, and a formal meeting was arranged later to further discuss the study objectives and protocol, sign the informed consent, and start the pre-fasting baseline measurements. All of the study participants completed the study to the end, with 100% compliance with the study requirements. This was driven by the enthusiasm to know how observing Ramadan fasting affects their body weight, metabolic, and other health measurements. Those who expressed willingness to participate were asked to visit the University Hospital Sharjah for a meeting. This meeting provided information about the study objectives and protocol and allowed examination of participants’ medical status and eligibility. Inclusion criteria were as follows: metabolically healthy obese (a subgroup of obese individuals who have a lower risk for cardiometabolic abnormalities [28] female or male adults (aged 18–60 years) who had not previously been diagnosed with any chronic or metabolic disease.

Those with a history of cardiovascular or endocrine disease and diabetes mellitus, along with pregnant women, were excluded. Those who took medication one week prior to Ramadan had bariatric surgery in the last 6–9 months or had engaged in a weight-reducing regimen 1 month before the commencement of Ramadan were also excluded. The daily fasting duration in this study was approximately 15 h. Obese or overweight adult male or female Muslims with body mass index (BMI) ≥ 25 kg/m^2^, who expressed their willingness to observe Ramadan fasting, were included. The Research and Ethics Committee of the University of Sharjah (REC-16-05-11-01) approved this study. All participants provided signed consent forms before starting this study.

### 2.3. Anthropometric Assessment

Waist circumference (WC), body height, and weight were measured using standardized techniques [29,30]. Height, without shoes, was measured using a stadiometer (Seca GmbH and Co., Hamburg, Germany, Model 217), while weight, without excess clothes, was measured using a body weight scale (Detecto, Webb City, MO, USA). WC was measured at the umbilicus, with participants in a standing position and breathing normally. BMI (kg/m^2^) was calculated accordingly. We measured blood pressure (both diastolic (DBP) and systolic (SBP)) while participants were in a sitting position using a digitalized monitor positioned in the upper arm (Omron Healthcare, Japan Model, BP 742 N).

### 2.4. Biochemical Assessment

Venipuncture blood sampling of 10 milliliters (mL) was undertaken for all participants 8–10 h after fasting (approximately between 11 a.m. and 1 p.m.). Within 2–3 h of blood sampling, whole blood samples were centrifuged, separated, and the collected serum samples were kept frozen in a deep freezer (at −80 °C). We used automated clinical chemistry analyzing machine (Adaltis, Pchem 1, Rome, Italy) to measure fasting glucose and lipid profile compartments (TC, LDL, HDL, and TG). ELISA kits (Elabscience, Houston, TX, USA) were used to measure fasting insulin, adiponectin, and leptin. Serum levels of PCSK9 were also measured using ELISA kits (Aviscera Bioscience Inc., Santa Clara, CA, USA). The insulin resistance marker (HOMA-IR) was calculated.

### 2.5. Dietary Intake Assessment

Assessment of food intake was performed using the 24 h food recall technique. Food intake information was collected for three days (one weekend day and two weekdays) before and during Ramadan by trained dieticians. Food models were used to assist participants to remember the proximity of consumed food portion sizes; total caloric intake and macro-and micronutrient intakes were assessed using Food Processor version 10.6 (ESHA Research, Salem, OR, USA).

### 2.6. Physical Activity Level

The Dietary Reference Intakes classification for general physical activity levels was used to assess participants’ levels of physical activity [31]. This classification depends on the general physical exercise pattern. Participants were considered to be highly active if they performed at least two hours/day of moderate-intensity physical exercise or one hour of vigorous exercise in addition to daily living activities. Participants were considered to be moderately active if they performed more than one hour/day of moderate-intensity exercise in addition to daily living activities. Participants who performed 30 min to 1 h/day of moderate-intensity physical exercise in addition to daily living activities were considered to have low activity. Finally, participants who performed daily living activities without other physical exercise were considered sedentary [31].

### 2.7. Statistical Analysis

Statistical analysis was conducted using SPSS version 17, and reported by applying the Strengthening the Reporting of Observational Studies in Epidemiology (STROBE) guidelines [32]. Change in PCSK9 levels between pre-fasting (baseline) and during fasting was considered the primary outcome. Sample size calculation indicated that a sample of 51 participants would provide 80% power, using a two-tailed, paired-samples *t*-test, with α = 0.05, to be able to find a significant difference of 5% in serum PCSK9 between baseline and during Ramadan fasting. We estimated a dropout rate of 10%. Therefore, a total of 57 participants was targeted. Using a Shapiro–Wilk test, non-parametric tests were applied after assessing the normality distribution of the data.

Categorical variables were expressed as a percentage and frequency of occurrence (for sociodemographic data: sex, marital status, nationality, educational level, and physical activity level). The median and interquartile range (IQR) were used to report non-normally distributed continuous variables (i.e., age, anthropometric, biochemical, and dietary data). Wilcoxon signed-rank tests were used to compare the changes in the studied variables before and during fasting. Linear regression was applied to determine the association between the changes in the dependent variable (PCSK9) before and at the end of Ramadan and the independent variables (FBG and leptin). All data were tested at the level of significance of *p* < 0.05.

## 3. Results

In total, 57 adults were recruited. and only 55 participants (22 females and 33 males) finished this observational prospective cohort study. The median (IQR) age was 35 (22) years, with a range of 20–59 years. Before Ramadan, fasting the SBP and DBP for all participants were 123.2 (10) and 71 (12) mmHg, respectively. Females had SBP of 123.2 (11) mmHg and DBP of 71 (12) mmHg, whereas males had SBP and DBP of 125 (14.5) mmHg and 71 (13.5) mmHg, respectively. The majority of participants were male (60%), and most participants were married (85%), held an undergraduate degree (80%), and were sedentary (95%) and from non-Gulf Cooperation Council (GCC) countries (Table 1).

We observed significant increases in serum levels of PCSK9 (median (IQR): 302 (106) ng/mL, *p* = 0.002), insulin (12.6 (12.1) ng/mL *p* = 0.002), leptin (1685 (1862) ng/mL, *p* < 0.001), and HOMA-IR (3.01 (3.05), *p* = 0.003) in comparison to the pre-fasting values. Conversely, there were significant reductions in BMI (30.2 (5.4) kg/m^2^, *p* < 0.001), WC (95 (10) cm, *p* = 0.001), SBP (123.2 (10) mmHg, *p* = 0.02), DBP (71 (12) mmHg, *p* = 0.05), TC (187.3 (33) mg/dL, *p* = 0.001), HDL-C (44.6 (12) mg/dL, *p* = 0.005), TG (94.2 (69) mg/dL, *p* < 0.001), and adiponectin (24.1 (13.8) μmol/mL, *p* = 0.001) compared to the pre-fasting measurements. No statistically significant changes were detected in FBG (91.5 (10) mg/dL, *p* = 0.05) or LDL-C (119 (44) mg/dL, *p* = 0.56) (Table 2).

Significant increases were reported in the intakes of total carbohydrates (median (IQR): 222.7 (44) g/d, *p* = 0.02), total water (1151 (509) ml/d, *p* < 0.001), vitamin C (54.33 (21.4) mg/d, *p* = 0.05), and omega-3 fatty acids (0.43 (0.1) mg/d, *p* = 0.002) at the end of Ramadan compared with pre-fasting. In contrast, there were significant decreases in the intake of *trans-*fats (0.83 (0.2) mg/d, *p* < 0.001) and lycopene (1173 (0.0) µg/d, *p* = 0.002) during Ramadan compared with pre-fasting (Table 3).

Before fasting, PCSK9 exhibited a highly significant (*p* < 0.001) moderate positive correlation with FBG (*r* = 0.40), and weak positive correlations with TG (*r* = 0.28) and HOMA-IR (*r* = 0.35). However, at the end of Ramadan fasting, PCSK9 only demonstrated a significant, but weak, positive correlation with leptin (*r* = 0.30, *p* < 0.05) (Table 4).

Table 5 shows the relationships between intakes of different nutrients before and at the end of Ramadan and the PCSK9 levels. There was a significant, but weak, positive correlation between PCSK9 serum levels and cholesterol intake (*r* = 0.274, *p* < 0.05) at the end of Ramadan fasting.

Linear regression analysis signified that, after adjusting for sex, age, physical activity, and BMI, both FBG and leptin levels before fasting were significantly associated with PCSK9 (B = 2.79, 95% CI: 1.26–4.31, *p* = 0.001 and B = 0.02, 95% CI: 0.001–0.303, *p* = 0.049, respectively). However, at the end of Ramadan fasting, only leptin demonstrated a significant association with PCSK9 (B = 0.013, 95%CI: 0.002–0.042, *p* = 0.026) (Table 6).

Considering the sex differences in PCSK9 levels between males and females before and after Ramadan fasting, the serum levels of PCSK9 of both sexes were not significantly different before Ramadan (285 (143.7) vs. 310 (84.5) ng/mL, *p* = 0.44). Similarly, at the end of Ramadan, there was no significant difference (347 (137) vs. 376 (147) ng/mL, *p* = 0.36). Likewise, females did not show significant changes in the serum levels of PCSK9 before and at the end of Ramadan (285 (143.7) vs. 347.2 (137) ng/mL, *p* = 0.08), while males had a significant increase in their serum levels of PCSK9 at the end of Ramadan (310 (84.5) vs. 376 (147) ng/mL, *p* = 0.0.009) when compared to their pre-fasting levels (Table 7).

## 4. Discussion

We measured plasma levels of PCSK9 for the first time to examine how this metabolic marker changed with RIF, making this the first study to describe changes in PCSK9 following exposure to RIF, as a form of non-pharmacological, dietary-modification-based intervention in human subjects. The leading findings of the present study were elevated levels of PCSK9, leptin, insulin, and HOMA-IR, and decreased levels of TC, HDL-C, TG, and adiponectin, during RIF among metabolically healthy obese people. A previously published work demonstrated that PCSK9 levels decreased with fasting [26] and caloric restriction [33]. However, we reported that plasma levels of PCSK9 increased by 22.1% at the end of RIF. These elevated levels of PCSK9 could be attributable to the diet presented in terms of the significant increases in total carbohydrate and total sugar intakes during the fasting month. Such increased intakes are associated with disturbing changes in the secretion of adipokines, such as decreased adiponectin and increased leptin [34]. Furthermore, leptin reduces the hepatocytes’ LDLR levels through the PCSK9 pathway, indicating the essential role of PCSK9 as a target molecule in obesity and dyslipidemia [35]. Moreover, the positive association between leptin and PCSK9 was shown only in healthy males when the BMI was <25 kg/m^2^. Additionally, on the molecular level, the PCSK9 promoter activity is enhanced in response to leptin and resistin adipokines through the contribution of the sterol regulatory element motif. In addition, they identify STAT3 as another activation molecular regulator in the resistin- and leptin-mediated transcription of PCSK9 [36]. Researchers in a previous work found that higher leptin levels significantly suppressed LDLR in HepG2 cells and increased PCSK9 expression [35], suggesting that leptin plays a role in regulating PCSK9 expression and hepatic LDLR, which could explain the mechanism responsible for dyslipidemia in obesity [35]. Such findings are supported by another work that revealed that adipokines (e.g., leptin) upregulate the expression of the PCSK9 protein [37].

The observed increase in PCSK9 levels at the end of Ramadan fasting was consistent with previous findings that lifestyle modifications for 1 year were associated with increased PCSK9 levels and related to visceral fat mobilization [38]. Visceral fat reduction upon RIF was also observed in the same group examined in the present study, as reported elsewhere [9]. This finding suggests that body fat distribution and visceral fat localization might affect circulating PCSK9 levels. Lastly, likely, the surprising results regarding the increase in PCSK9 levels with RIF may be because the study population was mainly people with obesity.

However, this finding contradicts those of Arsenault et al. [39], who reported that PCSK9 was not correlated with the distribution of body fat, while lifestyle behavioral modifications had minor effects on circulating PCSK9 levels in dyslipidemic men with abdominal obesity. Those authors explained their findings by suggesting that the relationship between PCSK9 and insulin resistance may occur through mechanisms that are independent of body fat distribution.

It is worth mentioning that PCSK9 is integral to lipid metabolism, and must not be principally branded as a bad, harmful marker. PCSK9 appears to have a beneficial effect, and (to some extent) is required to ensure an adequate lipid supply, as suggested by previous findings that long-term physical activity leads to a remarkable reduction in LDL-C levels, with a simultaneous remarkable increase in PCSK9 levels [40,41]. An inverse relationship between physical exercise and PCSK-9 [41] may help to explain the increased levels of PCSK9 during RIF, as fasting people tend to exhibit less activity than during non-fasting periods [42].

Ramadan fasting is characterized by a unique pattern of dietary intake, with major differences having been reported between Ramadan and the rest of the year [43]. People in the UAE, as in other Gulf countries, show a high intake of calories, protein, fats, and carbohydrates during Ramadan [4,9]; this type of diet could influence plasma PCSK9 levels. Research suggests that high-fat or high-protein foods do not change PCSK9 levels, whereas a fructose-rich diet tends to upregulate PCSK9 mRNA expression and elevate PCSK9 levels in humans [44] and animals [45,46].

Costet et al. (2006) indicated that PCSK9 expression is influenced by the nutritional status and serum levels of insulin. The present study showed that both blood glucose and insulin levels were elevated during Ramadan. Glucose increased by 7% and insulin by 63.5%, which ultimately increased the HOMA-IR by more than 50%. This could be attributed to the types of meals that people from different nationalities in the UAE usually eat at *Iftar* (post-sunset meal), which is often rich in carbohydrates (sugars) and fats [9,47]. This is supported by evidence that acute high fructose intake is linked to increased plasma PCSK9 levels in healthy subjects, independent of cholesterol synthesis [48].

The increased levels of insulin and HOMA-IR at the end of Ramadan observed in the current work are consistent with previous works on healthy adult subjects [49,50], though the meta-analysis on HOMAI-IR in 349 healthy subjects revealed a slight decrease in insulin resistance markers at the end of Ramadan in comparison with the pre-fasting levels [14]. These two metabolic changes are consistent with one another and could be explained by the excessive consumption of simple sugars during the nighttime, along with the drastic changes in circadian rhythms presented in terms of the shift in sleep time observed by many people during Ramadan [50,51].

In our previous work, serums levels of insulin, HOMA-IR, leptin, and adiponectin were systematically meta-analyzed in 3134 healthy subjects [14]. Our meta-analysis revealed that RIF was associated with improvement in some glucometabolic markers in healthy subjects, i.e., slightly increased adiponectin with slightly decreased leptin and HOMA-IR, and a slight increase in serum insulin in response to the excessive sugar intake during Ramadan’s night hours.

Applying a high-fructose diet, increased PCSK9 and insulin resistance were correlated, which is typically consistent with the increased HOMA-IR and insulin levels during Ramadan fasting [48]. Results for the same group of participants during the same month of fasting as used in the present study showed a significantly higher total sugar intake at the end of Ramadan compared with pre-fasting intake [9], which was confirmed later by a year-round comparative study carried out in Lebanon [43]. Results of this current work also demonstrated that PCSK9 levels were correlated with HOMA-IR and FBG, and the regression analysis exhibited that the FBG level was a good predictor of PCSK9 before the fasting month. However, this relationship was lost during Ramadan fasting, which may be attributable to the improved lipid profile and changes in sleep and dietary patterns [52].

Mechanisms explaining nutrient regulation of serum and hepatic PCSK9 may be observed in the impact of dietary nutrients on serum PCSK9 concentrations. Diets rich in monounsaturated fats (MUFAs), as well as n-6 and n-3 PUFAs, decrease PCSK9 levels. These fatty acids (particularly MUFAs and omega-3 PUFAs) increase AMPK, decrease inflammation, and activate the PPAR-α pathway, which may downregulate hepatic PCSK9 mRNA and activate SREBP2 expression. Higher intake of dietary cholesterol is also associated with decreased SREBP2 protein and an increased hepatic cholesterol pool and consequently downregulates the expression of hepatic PCSK9 mRNA [44]. The concurrent variable changes in dietary cholesterol and fatty acid intakes among our study participants made it difficult to ascertain the direct impact of dietary fats on PCSK9 and may suggest that other dietary, lifestyle or metabolic factors modulate PCSK9 during Ramadan.

PCSK9 is known to affect LDL levels [52,53]; however, we observed no changes in LDL at the end of Ramadan fasting despite the increased levels of PCSK9, which was consistent with previously published experimental work [52]. The lack of alignment between PCSK9 and LDL levels reported in this work could be explained by the fact that circulating PCSK9 levels are influenced by dietary, diurnal, and hormonal changes, while serum LDL cholesterol levels remain stable during the diurnal cycle [52]. Furthermore, excessive intake of fructose is thought to elevate serum PCSK9 levels via decreasing clearance through decreased expression of the LDLR protein. Fructose may also work to reduce serum PCSK9 concentration and hepatic PCSK9 mRNA through the pathways of the transcription factor sterol regulatory element-binding protein-1c (SREBP1c)-mediated signaling [44].

Several studies have indicated that PCSK9 may also affect the metabolism of other lipid components (e.g., TG), and a positive relationship between TG and PCSK9 levels has been demonstrated [54,55]. In this study, we also found that PCSK9 was positively correlated with TG before fasting; however, this relationship diminished during fasting.

Ramadan fasting showed variable effects on adiponectin, leptin, and insulin levels [14]. Both leptin and adiponectin are novel adipocyte-derived hormones that play a critical role in insulin sensitivity and glucose homeostasis in general. Leptin was the first adipokine described and is mainly secreted by adipose tissue [56]. Insulin has been reported to play a significant role in stimulating leptin production and secretion in adipose tissue [57]. Therefore, the high leptin levels in our participants could be ascribed to the significant increase in insulin levels. However, it appeared that leptin serum levels were closely related to the circulating PCSK9 before and at the end of Ramadan fasting

Adiponectin is secreted only by adipocytes [58], and exhibits insulin-sensitizing, fat-burning [59], inflammatory [60], and anti-atherogenic [61] effects. Sun et al. (2017) indicated that adiponectin receptor activation inhibited PCSK9 expression and concurrently provoked LDLR expression, thereby improving cholesterol metabolism. This could partially explain the anti-atherogenic features of adiponectin [62]. Putting this information together, if a fasting person has high dietary intakes of carbohydrates and fat, this results in high glucose and insulin levels (i.e., insulin resistance), increases leptin, decreases adiponectin, and increases circulating PCSK9. In turn, this is associated with a high risk of CVDs such as atherosclerosis [63], and possibly diabetes mellitus [64]. Therefore, to achieve the beneficial effects of fasting during Ramadan, proper dietary guidelines that restrict high-calorie diets—particularly high sugar intake—are needed.

Autophagosomes are delivered to lysosomes for degradation in a process called lipophagy and selectively sequester lipid-droplet-stored lipids. This was first observed in hepatocytes and represents a fundamental insight into how lipid metabolism controls cellular pathophysiology and physiology. PCSK9 may regulate lipid levels by affecting lipophagy [65]. Several studies have indicated that fasting interferes with the lipophagy process; prolonged fasting was found to promote lipophagy, as revealed by a list of transcription factors, whereas feeding suppressed lipophagy [65]. Therefore, prolonged fasting and starvation may reduce PCSK9 levels. This effect is not expected to be evident during RIF, because it involves alternating periods of feeding and fasting over 24 h (15/9 h during the year of the present study), with ad libitum food intake during the night hours. The lack of starvation and prolonged fasting during Ramadan was evident in the genetic expression of the sirtuins *SIRT1* and *SIRT3,* which play a critical role in restoring homeostasis during stress responses such as starvation and prolonged fasting, which is not the case during RIF [66].

Emerging evidence suggests that RIF has health-improving effects that may decrease the risk of developing cardiometabolic disorders [15], such as weight reduction [12], improved lipid profile, and glycemic control [8,14], and reduced oxidative stress and inflammatory markers [10]. While the significant weight loss reported at the end of Ramadan was expected to be positively reflected in PCSK9 levels, no effect on body weight loss was observed among fasting people. This finding was consistent with a prospective observational study involving 35 obese subjects, which showed that short-term weight loss did not significantly impact PCSK9 levels [67].

RIF is accompanied by changes in daily lifestyles and routines, which variably affect the markers of cardiometabolic diseases according to various factors, such as fasting duration, geographical location, and season [68]. Therefore, well-designed, large-scale interventions that control for various interfering and confounding factors are necessary to comprehensively evaluate the influence of RIF on cardiometabolic markers [51]. Studies in different geographical locations, ethnic backgrounds, and socioeconomic environments are warranted for a more accurate study of the PCSK9 changes during Ramadan fasting.

Some limitations should be considered when interpreting the findings of the present study. First, the lack of non-fasting control subjects from the same genetic and ethnic backgrounds means that it is difficult to generalize the study results to all people fasting during Ramadan. This was because, in most Muslim countries, it is hard to find non-fasting volunteers during Ramadan—particularly among healthy subjects without jurisprudential excuses. The lack of generalizability and reduced homogeneity of the study sample is another limitation of the followed convenience sampling technique [69]. Furthermore, physical activity was measured subjectively rather than objectively during this study, and could potentially have been biased. Nutritional intake was assessed with a memory-based technique (24 h recall method), and therefore may not reflect the actual food intake during the fasting month. Finally, this study did not test for changes in sleep quantity and quality or essential circadian rhythm hormones (such as melatonin and cortisol) that have metabolic impacts during Ramadan.

## 5. Conclusions

The results of this study provide essential information on how PCSK9 changes during RIF in overweight/obese subjects. The PCSK9 serum levels increased markedly at the end of Ramadan fasting, which may be attributable to complex relationships between PCSK9, insulin resistance, and the dysregulation of adipokine secretion. Such relationships are likely to be affected by dietary patterns—particularly the increased sugar intake reported by the study participants during the holy month of Ramadan.

## Figures and Tables

**Table 1:** Participants’ sociodemographic characteristics (*n* = 55).

Characteristic			*n* (%)
Age (Years)			35 (22) * [20,21,22,23,24,25,26,27,28,29,30,31,32,33,34,35,36,37,38,39,40,41,42,43,44,45,46,47,48,49,50,51,52,53,54,55,56,57,58,59] **
Sex			
		Male	33 (60)
		Female	22 (40)
Nationality			
		UAE and other GCC countries	3 (5.5)
		Non-GCC (Jordan, Syria, Palestine, Iraq, Egypt, Sudan, Tunisia)	52 (94.5)
Marital status			
		Married	47 (85)
		Single	8 (15)
Educational level			
		Basic education	5 (9)
		Undergraduate studies	44 (80)
		Postgraduate studies	6 (11)
Body mass index (BMI, kg/m^2^)		Overweight (25–29.9)	25 (45.5)
		Obese (≥30)	30 (54.5)
Blood pressure (mmHg) *			
	All	Systolic blood pressure (mmHg)	123.2 (10) *
		Diastolic blood pressure (mmHg)	71 (12) *
	Female	Systolic blood pressure (mmHg)	123.2 (11) *
		Diastolic blood pressure (mmHg)	71 (12) *
	Male	Systolic blood pressure (mmHg)	125 (14.5) *
		Diastolic blood pressure (mmHg)	71 (13.5) *
Physical activity			
		Sedentary	52 (95)
		Low activity	2 (3)
		Moderately active	1 (2)
		Highly active	-

* Median (interquartile range). ** Range (minimum-maximum). GCC, Gulf Cooperation Council; UAE, the United Arab Emirates.

**Table 2:** Changes in anthropometric and biochemical variables before and during Ramadan fasting.

Parameter	Before Ramadan *	At the End of Ramadan *	*p-*Value **
Body mass index (kg/m^2^)	30.2 (5.4)	29.7 (5.1)	<0.001
Waist circumference (cm)	95 (10)	92 (6)	0.001
Systolic blood pressure (mmHg)	123.2 (10)	119 (13)	0.02
Diastolic blood pressure (mmHg)	71 (12)	69 (11)	0.049
Fasting blood glucose (mg/dL)	91.5 (10)	98 (14.9)	0.05
Total cholesterol (mg/dL)	187.3 (33)	174.2 (40)	0.001
High-density lipoprotein cholesterol (HDL) (mg/dL)	44.6 (12)	40.6 (5.3)	0.005
Triglycerides (mg/dL)	94.2 (69)	74.8 (26)	<0.001
Low-density lipoprotein cholesterol (LDL) (mg/dL)	119 (44)	118.9 (32)	0.56
Serum insulin (ng/mL)	12.6 (12.1)	20.3 (14)	0.002
HOMA-IR	3.01 (3.05)	4.61 (3.5)	0.003
Adiponectin (µmol/mL)	24.1 (13.8)	18.4 (9.6)	0.001
Leptin (ng/mL)	1685 (1862)	4043 (6033)	<0.001
PCSK9 (ng/mL)	302 (106)	369 (121)	0.002

HOMA-IR: homeostatic model assessment for insulin resistance. PCSK9: proprotein convertase subtilisin/kexin type 9. * Median, (IQR: interquartile range). ** Using the Wilcoxon signed-rank test, significant change at *p <* 0.05.

**Table 3:** Changes in the nutrient intake before and at the end of Ramadan fasting month.

Nutrient	Before Ramadan *	At the End of Ramadan *	*p*-Value **
Total calories (kcal/d)	1745 (300)	1835 (684)	0.22
Total fats (g/d)	602 (109)	580 (326)	0.61
Protein (g/d)	69.36 (10.18)	68.62 (17.88)	0.30
Total carbohydrates (g/d)	222.7 (44)	251.2 (79)	0.02
Total sugars (g/d)	78.28 (24)	103 (43)	0.001
Saturated fats (g/d)	67.12 (12)	64.74 (36)	0.61
Monounsaturated fats (g/d)	13.93 (3.3)	13.81 (10.2)	0.30
Polyunsaturated fats (g/d)	6.98 (1.3)	9.2 (7)	0.44
*trans-*fats (mg/d)	0.83 (0.2)	0.54 (0.4)	<0.001
Cholesterol (mg/d)	227 (48)	217 (81)	0.25
Vitamin C (mg/d)	54.33 (21.4)	72.37 (45.9)	0.05
Beta-carotene (µg/d)	732 (499)	644 (512)	0.58
Omega-3 fatty acids (mg/d)	0.43 (0.1)	0.5 (0.7)	0.002
Omega-6 fatty acids (mg/d)	4.29 (2.1)	4.69 (3.9)	0.47
Lycopene (µg/d)	1173 (0.0)	822 (789)	0.002
Selenium (µg/d)	52.53 (8.2)	60.26 (26.7)	0.81
Vitamin E (mg/d)	3.75 (1.8)	3.5 (2.5)	0.26
Total water (ml/d)	1151 (509)	1821 (772)	<0.001

* Median, (IQR: interquartile range). ** Using the Wilcoxon signed-rank test, significant change at *p <* 0.05.

**Table 4:** Correlations between PCSK9 and studied variables before and at the end of Ramadan fasting.

Variable	Before Ramadan (*r*)	At the End of Ramadan (*r*)
Body mass index	0.10	0.04
Waist circumference	0.10	−0.007
Systolic blood pressure	0.10	−0.08
Diastolic blood pressure	0.10	−0.10
Fasting blood glucose	0.40 **	0.17
Total cholesterol	0.06	−0.10
HDL cholesterol	0.20	−0.07
Triglycerides	0.28 **	−0.01
LDL cholesterol	−0.49	−0.13
Serum insulin	0.16	−0.05
HOMA-IR	0.35 **	−0.02
Adiponectin	−0.068	0.176
Leptin	0.042	0.30 *

Using Pearson’s correlation coefficient, significant at * *p* < 0.05, ** *p* < 0.001. HOMA-IR: homeostatic model assessment for insulin resistance; HDL: high-density lipoprotein; LDL: low-density lipoprotein.

**Table 5:** Correlations between PCSK9 and different nutrients before and at the end of Ramadan fasting.

Nutrient	Before Ramadan (*r*)	At the End of Ramadan (*r*)
Total calories (kcal/d)	−0.160	0.097
Protein (g/d)	−0.190	0.113
Total carbohydrates (g/d)	−0.125	0.024
Total sugars (g/d)	0.064	0.018
Total fats (g/d)	−0.079	0.159
Saturated fats (g/d)	−0.145	0.152
Monounsaturated fats (g/d)	−0.101	0.178
Polyunsaturated fats (g/d)	−0.021	0.096
*trans*-fats (mg/d)	−0.037	0.172
Cholesterol (mg/d)	−0.167	0.274 *
Vitamin C (mg/d)	−0.119	0.026
Beta-carotene (µg/d)	−0.041	0.164
Omega-3 fatty acids (mg/d)	0.007	0.245
Omega-6 fatty acids (mg/d)	−0.027	0.147
Lycopene (µg/d)	−0.056	0.083
Selenium (µg/d)	−0.063	0.225
Vitamin E (mg/d)	0.002	0.129
Total water (ml/d)	−0.047	−0.217

* Using Pearson’s correlation coefficient, the significant correlation at *p* < 0.05.

**Table 6:** Linear regression analysis using PCSK9 as a dependent variable and fasting blood glucose and leptin as independent variables.

	Before Ramadan								At the End of Ramadan							
	Unadjusted				Adjusted *				Unadjusted				Adjusted *			
Independent Variable	B	95% CI		*p*-Value	B	95% CI		*p*-Value	B	95% CI		*p* Value	B	95% CI		*p*-Value
		U	L			U	L			U	L			U	L	
Fasting blood glucose	2.79	1.26	4.31	0.001	2.65	0.97	4.33	0.003	1.50	−1.00	4.00	0.23	1.55	−1.28	4.38	0.27
Leptin	0.02	0.001	0.03	0.049	0.03	0.005	0.053	0.018	0.013	0.002	0.024	0.026	0.014	0.002	0.026	0.025

CI: confidence interval. PCSK9: proprotein convertase subtilisin/kexin type 9. * Adjusted for age, sex, body mass index, and physical activity.

**Table 7:** Changes in the serum levels of PCSK9 (ng/mL) before and at the end of Ramadan fasting, according to sex.

Sex	Before Ramadan *	At the End of Ramadan *	*p*-Value ^$,^**
Females	285 (143.7)	347.2 (137)	0.08
Males	310 (84.5)	376 (147)	0.009
*p*-Value ^§,^**	0.44	0.36	

* Median (IQR: interquartile range). ** Using the Wilcoxon signed-rank test. ^$^ Intragroup comparison of before and at the end of Ramadan. ^§^ Within sex.

## Data Availability

Data are available upon request from the corresponding author.
